# Supplementary material for: Polyketide synthase gene clusters in the ericoid mycorrhizal fungus Oidiodendron maius: genome-wide prediction, expression profiling, and functional characterization of knockout mutants
Source: Front Plant Sci. 2026 Sep 16;17:1924430. doi: 10.3389/fpls.2026.1924430 (PMC13623903; doi:10.3389/fpls.2026.1924430)
Supplement: Supplementary file 1 [file SupplementaryFile1.zip › Supplementary Materials/Supplementary Folder 1.zip!/Oidma1_AssemblyScaffolds_Repeatmasked/knownclusterblast/cluster55/jgi.p_Oidma1_36230_mibig_hits.html]

| MIBiG Protein | Description | MIBiG Cluster | MiBiG Product | % ID | % Coverage | BLAST Score | E-value |
| --- | --- | --- | --- | --- | --- | --- | --- |
| AGO86659.1 | equisetin\_enoylreductase | BGC0001255 | Equisetin\_biosynthetic\_gene\_cluster | 46 | 98.1818181818 | 327 | 1.5e-89 |
| ABA02243.1 | dehydrogenase | BGC0000098 | Monacolin\_K\_biosynthetic\_gene\_cluster | 40 | 98.961038961 | 229 | 7.4e-60 |
| AAD34554.1 | enoyl\_reductase | BGC0000089 | Lovastatin\_biosynthetic\_gene\_cluster | 40 | 98.4415584416 | 228 | 9.7e-60 |
| CCT72378.1 | related\_to\_C.carbonum\_toxD\_protein | BGC0001305 | Fujikurins\_biosynthetic\_gene\_cluster | 34 | 97.6623376623 | 193 | 4.5e-49 |
| CAL69596.1 | hypothetical\_protein | BGC0001049 | Tenellin\_biosynthetic\_gene\_cluster | 35 | 99.7402597403 | 191 | 2.2e-48 |
| CAA63129.1 |  | BGC0000370\_c4 | HC-toxin\_biosynthetic\_gene\_cluster | 33 | 79.7402597403 | 127 | 4e-29 |
| EHA28239.1 | hypothetical\_protein | BGC0001143 | Azanigerone\_biosynthetic\_gene\_cluster | 30 | 64.1558441558 | 78 | 2.1e-14 |
